# The Role of Virtual Cystoscopy, after Multidetector Computed Tomography Imaging Reconstruction without the Use of Contrast Medium, in the Diagnosis and Evaluations of Bladder Tumors: Preliminary Study

**DOI:** 10.1155/2014/923958

**Published:** 2014-04-02

**Authors:** Kyriaki Kalokairinou, Achilles Ploumidis, Theodoros Kalogeropoulos, Lampros Vlachos, Kyriakos Stringaris, Ageliki Tavernaraki, Anastasios Thanos, Xenofon Papacharalampous, Vasilios Koutoulidis, Julien Letendre, Olivier Traxer, Athanasios Gouliamos

**Affiliations:** ^1^Department of Radiology, KAT Hospital, 2 Nikis Street, Kifisia, 14561 Athens, Greece; ^2^Department of Urology, Athens Medical Center Hospital, Athens, Greece; ^3^Department of Urology, AG Savvas Anticancer Hospital, Athens, Greece; ^4^Department of Radiology, Aretaieion University Hospital, Athens Medical School, Athens, Greece; ^5^Department of Radiology, Henry Dunant Hospital, Red Cross Foundation, Athens, Greece; ^6^Department of Radiology, AG Savvas Anticancer Hospital, Athens, Greece; ^7^Department of Urology, Tenon University Hospital, Paris, France

## Abstract

*Introduction*. Although conventional cystoscopy is considered to be the gold standard for diagnosis and follow-up of bladder tumors, it remains an invasive and costly procedure. With the advent of the multidetector CT (MDCT) scanners supported by specialized software virtual cystoscopy (VC) is possible. We assess the role of VC in diagnosing and evaluating bladder lesions. *Materials and Methods*. Between September 2010 and October 2011, 25 consecutive patients with cystoscopically confirmed bladder tumor underwent VC. The radiologists involved in this prospective study were blinded to the exact findings. After draining any residual urine with a catheter, the bladder was retrogradely insufflated with 200–600 cc of air. No intravenous or intravesical contrast was used. MDCT scan was performed in supine and prone positions and three-dimensional reconstruction of the urinary bladder was performed. *Results*. The examination was well tolerated by all patients with no complications. In total, 43 lesions were detected both with conventional cystoscopy and VC. Tumor size measured by CT ranged from 3 to 80 mm in diameter. The pathological report revealed noninvasive transitional cell carcinomas in all cases. *Conclusion*. VC has promising results in detecting exophytic bladder lesions. In the future it could be part of the diagnostic algorithm for bladder tumors.

## 1. Introduction

Rigid and flexible cystoscopy is considered the gold standard for diagnosis and follow-up of bladder cancer [1, 2]. However, disadvantages such as patient discomfort, risk of urinary sepsis, and possible injury of the urethra with subsequent urethral stenosis render this procedure invasive not to mention expensive considering the long follow-up of bladder cancer patients [1, 3–5].

On the other hand, recent advancements in CT technology with rapid image acquisition due to multidetector CT (MDCT) scanners combined with powerful three-dimensional software have led to the development of virtual reality imaging [1, 2]. The technique has already been successfully implemented in other hollow organs such as the colon, the bronchus, and the stomach [3–6], while it has proven to be a useful complementary tool for the evaluation of the entire urinary tract [7–12]. The aim of our study is to assess the role of virtual cystoscopy (VC) with MDCT in diagnosing and evaluating bladder lesions demonstrating potential advantages and pitfalls.

## 2. Materials and Methods

### 2.1. Study Population

Between September 2010 and October 2011, 25 consecutive patients (20 men and 5 women), median age 61 years (range: 42–81), with a cystoscopically confirmed bladder tumor, underwent VC with MDCT scanning. All patients recruited in this prospective study presented initially at the department of urology with painless gross hematuria for the first time and no other previous urological history. After typical evaluation that included cystoscopy during which bladder tumor was documented the patients were referred to the department of radiology for VC. The time interval between conventional cystoscopy and VC ranged from one to two days. The radiologists involved in this survey, apart from knowing the existence of at least one bladder lesion, were blinded to the exact cystoscopic results (number, size, location, morphology, and type of tumor). Patients taking antiplatelet and anticoagulant therapy were excluded from the study, while written informed consent was obtained from each patient and the study was approved by the institutional review board.

### 2.2. VC Technique

Any residual urine was drained through a 14-F Foley catheter, which was then used for retrograde insufflation of the urinary bladder with 200–600 cc (mean: 350 cc) of air depending on patient's tolerance, while the catheter was left in place and was clamped. Then, the patient was placed in a supine position and a scout view was done to locate the urinary bladder and confirm its adequate distension. MDCT scan was performed (Aquilion 64, Toshiba Medical System Europe) with the following parameters: 0.5 mm thickness, 120 kV, 163 mA, and 1000 msec. This technique of retrograde insufflation of the urinary bladder with air has been previously described for the successful reconstruction of the organ [13]. In this position, images were taken initiating the sequence from 2 cm above and finalizing the exam 2 cm below the urinary bladder. Neither intravenous contrast nor sedation was used during CT scanning. The radiation dose was evaluated using the Tsalavoutas method [14], while the CT dose index was calculated for each patient and varied from 8 to 10 mGy with a sum of effective dose of 5.92 mGy. Subsequently, the patient was turned in prone position and a helical CT scan of the urinary bladder was repeated with the same parameters. The reason for the MDCT in prone position was to increase the accuracy in the 3D reconstruction of the bladder and to eliminate artifacts from residual urine. Each examination was performed using a single-breath-hold technique, while the radiation threshold was optimized at 500 HU. The total scanning time ranged from 3 to 5 seconds. Finally, images with 0.5 cm intervals were reconstructed by using the minimal field of view measured from the inner aspect of the centre of the pelvis.

**Figure 1:**
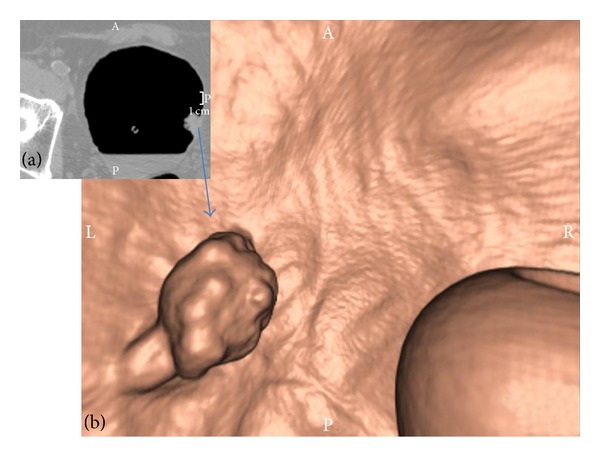
Exophytic lesion arising from the left side of the bladder wall. (a) MDCT image (transverse plane) and (b) VC image (after 3D reconstruction). The pathology report revealed a pTa TCC of the bladder. On the right side of the VC image (b) the balloon of the catheter can be noted.

**Figure 2:**
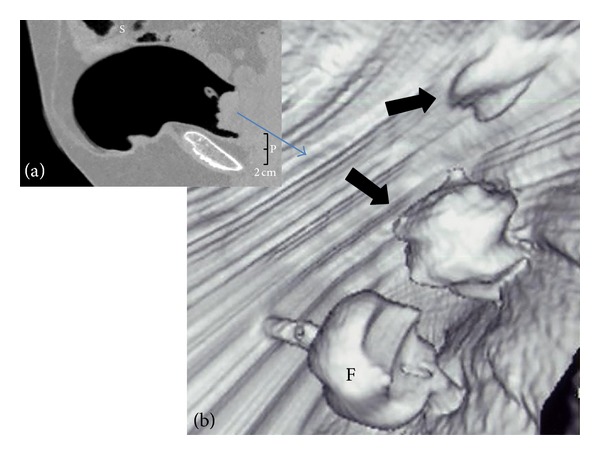
Exophytic lesion arising from the posterior side of the bladder wall. (a) MDCT image (sagittal plane) and (b) VC image (after 3D reconstruction). The pathology report revealed a pT1 TCC of the bladder.

**Figure 3:**
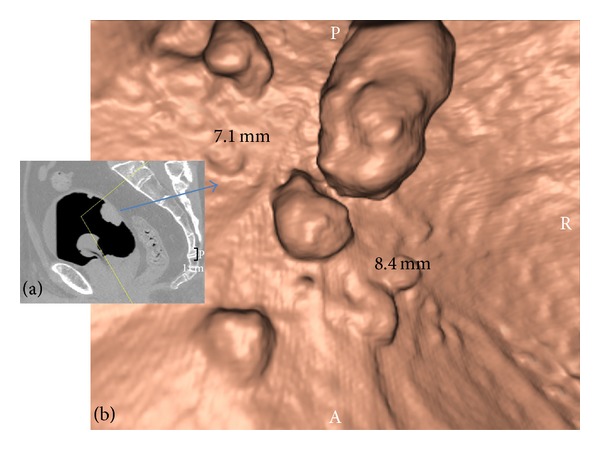
Multifocal exophytic lesions on the anterior and posterior wall of the bladder. (a) MDCT image (sagittal plane) and (b) VC image (after 3D reconstruction). The pathology report revealed a pT1 TCC of the bladder.

The DICOM imaging data was then downloaded to an independent computer workstation and was processed by an interactive intraluminal navigation software with a volume-rendering algorithm (Vitrea 5.2 fX 3.1, Vital Imaging Inc., CA, USA). This software allowed for a three-dimensional reconstruction of the urinary bladder using the conventional MDCT images. The reconstructed bladder could be viewed intraluminally in any angle giving the impression of virtually “flying” in the hollow organ (Figures 1, 2, and 3). Additionally, areas of interest could be magnified and marked, whereas the pictures viewed could be exported to PACS devices.

During the virtual cystoscopic study, the “camera” of the virtual cystoscope was placed in the centre of the bladder lumen and each quadrant of the 3D reconstructed bladder was carefully observed in a scrolling motion. When a lesion of the bladder mucosa was noted, it was magnified and fully evaluated from various angles. The number, size, location, and morphologic features of the lesions were evaluated using both virtual images, reproduced by the software, as well as the conventional transverse images obtained in the supine and prone positions. When a lesion was observed, it was characterized as focal polypoid when its intraluminal projection was greater than its base or sessile in case its base was significantly wider. Furthermore, when elevation of the bladder wall was detected without the presence of a discrete mass, it was documented as bladder wall thickening.

## 3. Results

The bladder catheter was successfully placed and bladder distension was adequate in all patients, while good quality images were obtained after three-dimensional reconstruction. The examination was well tolerated by all patients, and no complications or urinary tract infections occurred. The number, the site, and the size of the bladder lesions detected by VC were consistent with the findings from conventional cystoscopy. No false positive or negative results were reported with VC. Among the patients studied, 8 had more than one tumor. In total, 44 lesions in 25 patients were detected with both conventional cystoscopy and VC (Table 1). Tumor size measured by CT ranged from 3 to 80 mm in diameter with 29 larger than 5 mm and 15 being 5 mm or less. The pathological report revealed transitional cell carcinomas (TCC) in all cases. Men with bladder trabeculation due to BPH were more challenging in diagnosing small lesion of the epithelium compared to women that rarely presented with detrusor hypertrophy. The total time spent on study interpretation was approximately 5–7 minutes.

## 4. Discussion

Our study suggests that VC with the use of MDCT scanners and no contrast medium is possible in both men and women. The examination was well accepted by all patients with minimal discomfort and no complications occurred. With the help of modern software, high quality three-dimensional images were reproduced giving adequate information on the intraluminal morphology of the bladder.

In 1996, Vining et al. were the first to report the feasibility of VC in one healthy volunteer and two patients with biopsy-proven TCC of the bladder [15]. Based on a number of reports, VC emerged as a promising method in obtaining both intravesical and extravesical imaging information, with results comparable to that of conventional cystoscopy in the detection of bladder lesions [16–19]. Although low sensitivity rates were reported for lesions smaller than 1 cm, Kim et al. later on upgraded the sensitivity of the method by using a slice thickness of 1.25 mm [1]. MDCT scanners demonstrate advantages such as: the ability to cover extended anatomic volumes combined with the substantially reduced examination time, which is essential for reduction of motion artifacts. In addition to the above, the considerable reduction of section collimation, have all together revolutionized the imaging technology [20]. VC with MDCT enables a fast and detailed evaluation of the urinary bladder and pelvic region, with satisfactory results in detecting bladder lesions, especially small-sized exophytic tumors [2, 13].

As a minimally invasive procedure to detect exophytic lesions of the bladder mucosa, VC has several advantages over conventional cystoscopy. The volumetric imaging data obtained with helical CT is computer rendered to generate three-dimensional images and with commercially available software, intraluminal navigation through any hollow organ can be possible. The VC images can also be stored in a database facilitating lesion comparison during follow-up. Additionally, the size of a lesion can be measured easily and objectively [18]. Furthermore, detection of a tumor within a diverticulum is possible with the aid of software reconstruction tools even in cases where the narrow neck of the diverticulum could render cystoscopy very difficult [8]. Patients with a severe urethral stricture, who may be poor candidates for conventional cystoscopy, can safely undergo VC as far as a fine catheter can be placed in the bladder successfully. The indications may be extended to young children where cystoscopy is cumbersome as well as in patients with high risk of iatrogenic hemorrhage [1, 18, 21]. Finally, the use of transverse images during VC allows for evaluation of additional pelvic pathologies, extravesical metastases, or even clinically positive pelvic lymph nodes [1].

On the other hand, although conventional cystoscopy is considered the gold standard for diagnosis and follow-up of bladder tumors, it still remains an uncomfortable invasive procedure that involves inserting a flexible or rigid scope through the urethra in order to examine the bladder [1, 15, 19]. Another important issue is the financial aspect of cystoscopy for the health care system. Since patients with confirmed TCC should be vigorously monitored with cystoscopy for at least five years the accumulated cost rises significantly [22]. It is apparent that the need for a cost effective substitution of the present frequent follow-up method and the monitoring of patients is eminent [23].

There are several disadvantages to VC. The main limitation is the difficulty to detect flat and superficial lesions [2, 16–19]. Additionally, differential diagnosis of a TCC from a simple thickening of the mucosa due to fibrositis may be very difficult sometimes, especially when bladder distension is insufficient. Furthermore, the need for a second CT in prone position can be time consuming and the additional exposure for the patient can be an issue. Moreover, in the long run, if VC were to be used as a follow-up tool for bladder cancer, with the current technology of MDCT, the cumulative radiation for the patient needs to be considered. Some patients may find bladder distension with air annoying, while mixture of the contrast material with urine may in some cases result in imaging artifacts. In addition, possible scheduling problems may arise in a busy radiological department because of the repeated patient positioning and scanning required.

Our promising results of VC are limited by the fact that the number of patients included is small and our study is not randomized and controlled. Furthermore, the patients recruited for VC had cystoscopically proven bladder lesions and since the radiologists were not blinded to the existence of at least one bladder lesion, this could falsely increase the sensitivity of the method. Additionally, during conventional cystoscopy, no flat lesions were noticed and all patients had only exophytic tumors, which can justify our excellent results.

## 5. Conclusion

The results of VC are encouraging but still not sufficient enough to totally replace conventional cystoscopy for the screening, diagnosis, and follow-up of bladder lesions. With the advent of further technological and software refinements, this minimally invasive method could in the near future be a useful tool as part of the diagnostic or follow-up algorithm in combination with other molecular tests.

## Figures and Tables

**Table 1:** The clinical characteristics (age, gender) of the patients as well as the number, size (length of biggest tumor when multiple), and pathology of the tumors observed.

Patient number	Age	Gender	Tumor size (max)	Tumor number	Pathology
1	43	Female	<5 mm	1	TaG1
2	63	Male	>5 mm	1	TaG3
3	45	Male	>5 mm	2	T3b
4	76	Male	<5 mm	1	T3b
5	79	Male	>5 mm	1	T3b
6	62	Male	<5 mm	1	T3a
7	65	Male	<5 mm	3	T3
8	58	Male	>5 mm	1	T3
9	67	Male	<5 mm	1	T3
10	69	Male	>5 mm	1	T3a
11	76	Male	>5 mm	4	T3
12	49	Female	<5 mm	3	T3b
13	50	Female	>5 mm	1	T3
14	52	Female	>5 mm	1	T3
15	57	Male	>5 mm	1	T1
16	47	Female	>5 mm	4	T3
17	81	Male	>5 mm	1	T3b
18	66	Male	<5 mm	1	T3a
19	63	Male	>5 mm	1	T3a
20	65	Male	<5 mm	1	T3b
21	68	Male	>5 mm	6	T3a
22	77	Male	<5 mm	1	T3b
23	42	Male	>5 mm	1	T3a
24	64	Male	>5 mm	3	T3a
25	60	Male	<5 mm	2	T3b

## References

[1] Kim JK, Ahn JH, Park T, Ahn HJ, Kim CS, Cho KS (2002). Virtual cystoscopy of the contrast material-filled bladder in patients with gross hematuria. *The American Journal of Roentgenology*.

[2] Arslan H, Ceylan K, Harman M, Yilmaz Y, Temizoz O, Can S (2006). Virtual computed tomography cystoscopy in bladder pathologies. *International Brazilian Journal of Urology*.

[3] Inamoto K, Kouzai K, Ueeda T, Marukawa T (2005). CT virtual endoscopy of the stomach: comparison study with gastric fiberscopy. *Abdominal Imaging*.

[4] Lacasse Y, Martel S, Hébert A, Carrier G, Raby B (2004). Accuracy of virtual bronchoscopy to detect endobronchial lesions. *Annals of Thoracic Surgery*.

[5] Panebianco V, Osimani M, Lisi D (2009). 64-detector row CT cystography with virtual cystoscopy in the detection of bladder carcinoma: preliminary experience in selected patients. *Radiologia Medica*.

[6] Yun JY, Ro HJ, Park JB (2007). Diagnostic performance of CT colonography for the detection of colorectal polyps. *Korean Journal of Radiology*.

[7] Kagadis GC, Siablis D, Liatsikos EN, Petsas T, Nikiforidis GC (2006). Virtual endoscopy of the urinary tract. *Asian Journal of Andrology*.

[8] Prando A (2002). CT-virtual endoscopy of the urinary tract. *International Brazilian Journal of Urology*.

[9] Takebayashi S, Hosaka M, Kubota Y (2000). Computerized tomographic ureteroscopy for diagnosing ureteral tumors. *Journal of Urology*.

[10] Battista G, Sassi C, Schiavina R (2009). Computerized tomography virtual endoscopy in evaluation of upper urinary tract tumors: initial experience. *Abdominal Imaging*.

[11] Chou CP, Huang JS, Wu MT (2005). CT voiding urethrography and virtual urethroscopy: preliminary study with 16-MDCT. *The American Journal of Roentgenology*.

[12] Chou CP, Huang JS, Yu CC, Pan HB, Huang FD (2005). Urethral diverticulum: diagnosis with virtual CT urethroscopy. *The American Journal of Roentgenology*.

[13] Tsampoulas C, Tsili AC, Giannakis D, Alamanos Y, Sofikitis N, Efremidis SC (2008). 16-MDCT cystoscopy in the evaluation of neoplasms of the urinary bladder. *The American Journal of Roentgenology*.

[14] Tsalafoutas IA, Metallidis SI (2011). A method for calculating the dose length product from CT DICOM images. *The British Journal of Radiology*.

[15] Vining DJ, Zagoria RJ, Liu K, Stelts D (1996). CT cystoscopy: an innovation in bladder imaging. *The American Journal of Roentgenology*.

[16] Fenlon HM, Bell TV, Ahari HK, Hussain S (1997). Virtual cystoscopy: early clinical experience. *Radiology*.

[17] Narumi Y, Kumatani T, Sawai Y (1996). The bladder and bladder tumors: imaging with three-dimensional display of helical CT data. *The American Journal of Roentgenology*.

[18] Song JH, Francis IR, Platt JF (2001). Bladder tumor detection at virtual cystoscopy. *Radiology*.

[19] Tsili AC, Tsampoulas C, Chatziparaskevas N (2004). Computed tomographic virtual cystoscopy for the detection of urinary bladder neoplasms. *European Urology*.

[20] Flohr TG, Schaller S, Stierstorfer K, Bruder H, Ohnesorge BM, Schoepf UJ (2005). Multi-detector row CT systems and image-reconstruction techniques. *Radiology*.

[21] Lämmle M, Beer A, Settles M, Hannig C, Schwaibold H, Drews C (2002). Reliability of MR imaging-based virtual cystoscopy in the diagnosis of cancer of the urinary bladder. *The American Journal of Roentgenology*.

[22] Sylvester RJ, van der Meijden AP, Oosterlinck W (2006). Predicting recurrence and progression in individual patients with stage Ta T1 bladder cancer using EORTC risk tables: a combined analysis of 2596 patients from seven EORTC trials. *European Urology*.

[23] Botteman MF, Pashos CL, Redaelli A, Laskin B, Hauser R (2003). The health economics of bladder cancer: a comprehensive review of the published literature. *Pharmacoeconomics*.

